# Evaluation of Biomaterials in Periodontal Regeneration: A Literature Review

**DOI:** 10.7759/cureus.75618

**Published:** 2024-12-12

**Authors:** Vidya Pranathi, Rekha R Koduganti, Soumya Muthyala, Sai Pranavi Kanchanapally, Nitheeesha Muthyala, Vyshnavi Shingade

**Affiliations:** 1 Department of Periodontics, Panineeya Institute of Dental Sciences and Research Centre, Hyderabad, IND

**Keywords:** 3d scaffolds, biologicals, bone grafts, gtr, guided tissue regeneration, guided tissue regeneration (gtr) membrane, tissue engineering

## Abstract

The field of periodontal regeneration focuses on restoring the form and function of periodontal tissues compromised due to diseases affecting the supporting structures of teeth. Biomaterials have emerged as a vital component in periodontal regenerative therapy, offering a variety of properties that enhance cellular interactions, promote healing, and support tissue reconstruction. This review explores current advances in biomaterials for periodontal regeneration, including ceramics, polymers, and composite scaffolds, and their integration with biological agents like growth factors and stem cells. Specifically, biomaterials such as hydroxyapatite and bioactive glass provide scaffolding for cellular adhesion and proliferation, while synthetic and natural polymers offer flexibility and biocompatibility. Growth factors and bone morphogenic proteins (BMP) further support cell differentiation and tissue formation, enhancing clinical outcomes in periodontal defect repair. Moreover, stem cell integration with biomaterials, particularly the periodontal ligament stem cells (PDLSCs) and mesenchymal stem cells (MSCs), shows promise for complex tissue regeneration by stimulating targeted healing responses in periodontal tissue. Although clinical results are encouraging, challenges related to the selection of optimal biocompatible and bioactive materials and standardization of clinical protocols remain. This review examines the potential, limitations, and future directions for biomaterial-based strategies, highlighting the evolving role of these materials in achieving predictable and effective periodontal regeneration.

## Introduction and background

Periodontitis can severely damage tissues, leading to significant alveolar bone loss and gingival recession, often resulting in root cementum exposure [1]. Current treatments for periodontitis, such as scaling, root planing, and surgical interventions, focus on plaque removal and inflammation control. While these therapies aim to alleviate symptoms and halt disease progression, they do not restore the original attachment of periodontal tissues to teeth [2]. Treatment outcome is considered successful when new periodontal ligament fibers, new cementum, and new bone are laid down. The migration growth and attachment of periodontal ligament (PDL) cells to the root surface enable the maturation of a functional structure that integrates into freshly formed cementum. Similarly, progenitor bone cells must migrate, proliferate, and mature alongside the regenerating periodontal ligament. Thus, the attachment of healthy cells at the healing site is mandatory for true regeneration [3]. However, the selective proliferation of PDL cells poses a challenge. Guided tissue regeneration (GTR) membranes are available both as nonabsorbable and absorbable variants, and their proper selection and application improve the treatment outcome [4,5]. Though nonabsorbable membranes like polytetrafluoroethylene (PTFE) are biocompatible and possess excellent mechanical properties, their nature requires a second surgical protocol for their removal, which may cause infection and increase the chances of damage to the newly formed periodontal tissue [6]. Absorbable membranes, on the other hand, negate the need for a second surgery; however, these membranes, which are made of synthetic or natural polymers, have low mechanical strength, and their rate of degradation cannot be controlled precisely, limiting their application in regenerative procedures. Therefore, the choice of GTR membrane should be made taking into consideration all these factors [7]. Today, regenerative periodontal therapy continues to evolve, incorporating a combination of GTR, biological agents, hydrogels, and emerging technologies such as nanomaterials and gene therapy to enhance the predictability and effectiveness of treatment outcomes. A variety of nanomaterials, including nanoparticles (NPs), nanocapsules, nanocomposites, nanofibers, nanotubes, and nanosheets, have demonstrated promising results and show potential for rebuilding structures and restoring the functions of oral tissues [8]. This review aims to highlight the role of these newer technologies in periodontal regeneration.

## Review

Periodontal regeneration

Periodontal regeneration aims to fully restore the tissues destroyed by periodontal disease, including the alveolar bone, periodontal ligament, and cementum. Traditional periodontal therapies focus on halting disease progression and preventing further tissue destruction. However, periodontal regeneration goes beyond disease control, seeking to biologically reconstruct the original periodontal apparatus. The process of regeneration involves complex biological mechanisms, including cell migration, proliferation, differentiation, and formation of extracellular matrices. Achieving true periodontal regeneration requires addressing multiple tissue types that possess different regenerative capacities [9]. Melcher [10] stated that the epithelial cells proliferated very fast, and therefore, the chances of these cells migrating to the defect site were very high. If both periodontal ligament and bone have to be regenerated, then cells having the capacity to regenerate and maintain the alveolar part of the periodontium, namely cementum, periodontal ligament, and alveolar bone, should colonize the wound rather than connective tissue cells from the gingiva or bone alone. Caton and Greenstein [11] opined that the healing response following conventional periodontal procedures was by repair and elaborated on the important variables in periodontal wound healing, such as root surface alterations, progenitor cell populations, epithelial exclusion from the wound, reduced periodontium, and wound stabilization.

Guided tissue regeneration and barrier membrane

Guided tissue regeneration membranes used in regenerative treatment facilitate contact guidance, which allows for the timely migration of cells into the healing site, promoting regeneration [12]. Nonresorbable membranes that were initially utilized were expanded polytetrafluoroethylene (e PTFE). The limitation of these membranes was that extensive releasing incisions were required to remove these membranes after an optimal healing period due to tissue ingress. High-density polytetrafluoroethylene membranes (d PTFE) with a pore size of 0.3 microns were later introduced. These membranes could be easily removed, but they collapsed into the surgical site. This led to their replacement with titanium-reinforced, high-density polytetrafluoroethylene membranes. Nonresorbable membranes were soon discarded due to the risk of wound dehiscence and the requirement of a second surgery for their removal. This led to the advent of resorbable membranes that are primarily of two types. Those composed of polymers, namely, synthetic membranes, and those derived from collagen obtained from animal sources that are natural membranes. Barrier membrane technology has advanced in recent years to include functional membranes that include the incorporation of antibacterial agents, bioactive agents, growth factors, and a multilayered architecture to facilitate regeneration [13]. 

To prevent reinfection after membrane placement, the addition of materials to the membrane with antimicrobial properties has been preferred. An ideal example of such a material is chitosan, a natural, seashell-derived polymer with antibacterial, antifungal, bio-adhesive, and hemostatic effects [14]. Lee et al. reported that a chitosan membrane with grafted epigallocatechin-3-gallate and lovastatin showed a bactericidal effect on putative pathogens such as *Aggregatibacter actinomycetemcommitans*, *Prevotella nigrescens*, and *Porphyromonas gingivalis* [15]. Zhou et al. demonstrated that the scaffold composed of fish collagen/bioactive glass/chitosan composite nanofibers had antibacterial effects on *Streptococcus mutans*, leading to an effective regeneration of furcation defects in a dog model [16]. Bioceramics like hydroxyapatite, bioglass, and beta-tricalcium phosphate (β-TCP) have been widely used to improve the healing of alveolar bone in periodontitis [17]. The main advantage of bioceramic scaffolds over natural polymers is their remarkable osseoinductive and osseoconductive properties.

A study enrolled 20 patients who were equally split into two groups. In group I, the patients with intra-bony defects received β-TCP with a collagen membrane, whereas in group II, the patients with intra-bony defects received cultured gingival fibroblasts (GF) on the β-TCP scaffold with a collagen membrane. The clinical and radiological evaluation was done at baseline and six months. It was observed that there was a greater reduction in probing pocket depth and clinical attachment level gain in group II over group I. The radiological bone gain was also better in group II [18]. Hydrogels as scaffolds have shown good results in periodontal tissue regeneration, offering a biomimetic environment similar to natural biological tissues and the extracellular matrix [19]. Polymeric materials have the dual advantage of retaining and absorbing water and maintaining a solid structure due to the three-dimensional network of polymer chains. Excess in water content of these gels makes them soft with an ideal milieu for cell adhesion, proliferation, differentiation, and growth [20].

Pan et al. conducted a study on rats using an injectable hydrogel scaffold containing nano-hydroxyapatite. After four weeks of administration in alveolar defects, it was observed that the scaffold degraded completely, leading to significant alveolar bone regeneration without any untoward inflammatory events [21].

Bone grafts

Hard tissue grafts or substitutes are often placed into the debrided periodontal defect during surgery to stimulate tissue regeneration. The idea behind this is that bone formation will promote new attachment. Currently, a wide range of materials are available for regenerative periodontal therapy. They are broadly categorized as autografts, allografts, xenografts, and alloplasts. Autografts are collected from one site on the same individual and transplanted to a different location. While autografts provide the most osteogenic organic material for grafting, they have drawbacks such as donor site morbidity and limitations on the volume of graft material that can be harvested. Extraoral autografts like iliac grafts are not being used these days due to higher morbidity, risk of contamination, and increased surgical time. Moreover, its replacement rate may be unpredictable. Orsini et al. [22] compared calcium sulfate and autograft with bioabsorbable membranes and autograft in intra-bony defects and concluded that both materials were equally effective and led to short-term improvements in the clinical parameters assessed.

Jindal et al. [23] evaluated the effectiveness of intra-oral autogenous grafts versus decalcified allogenic bone matrix (DABM) in treating periodontal intra-bony defects. Thirty patients aged 30-50 years with paired defects were included. Either DABM or intraoral autogenous grafts were placed in each of the defects. Radiographical assessments at the third and sixth months showed both treatments resulted in bone fill, with the autogenous graft demonstrating significantly higher effectiveness compared to DABM at both time points.

Another study [24] was conducted to assess histologic healing following tooth extraction with ridge preservation using mineralized versus combined mineralized-demineralized freeze-dried bone allograft. The study proved that a combination of mineralized and demineralized allografts yielded better results in alveolar ridge preservation than when mineralized allografts alone were used.

Biologicals

Growth factors and signaling molecules are proteins that promote chemotaxis, proliferation, differentiation, extracellular matrix synthesis, and angiogenesis [25]. Though the biological functions of these factors vary widely, their selection as agents for regenerative therapy is based on their important roles in periodontal tissue synthesis and wound healing.

Platelet-Derived Growth Factor (PDGF)

Promotes wound healing by enhancing the activity of PDL cells, aiding collagen synthesis, and gingival fibroblast activity. Recombinant human PDGF (rh-PDGF) has become commercially available for use in the form of a growth-factor-enhanced matrix, GEM 21S (Lynch Biologics), which utilizes β-TCP as a scaffold [26]. The PDGF family is composed of four gene products capable of forming five different dimeric isoforms. These isoforms include PDGF-AB, -AA, -BB, -CC, and -DD variants. A recent meta-analysis conducted by researchers reported that treatment of periodontal osseous defects with rh-PDGF-BB resulted in significantly increased bone fill, lateral bone gain, clinical attachment gain, and probing depth reduction, compared to control groups utilizing modified minimally invasive surgical techniques or bone filler biomaterial alone (β-TCP) [27]. The current evidence, however, is inconsistent related to the use of rh-PDGF-BB in recession defects, sinus augmentation, and socket preservation procedures.

Bone Morphogenic Proteins (BMPs)

The bone morphogenic proteins BMP-2 and BMP-7 have shown great promise in periodontal regeneration, primarily by inducing osteogenesis and cementogenesis. BMP-2, known for its osteogenic properties, has been utilized extensively in periodontal therapy. rhBMP-2 is commercially available as an Infuse Bone Graft (Medtronic) and is FDA-approved for sinus augmentation and alveolar ridge preservation. A systematic review conducted by some researchers reported that sinus augmentation with autogenous bone resulted in significantly greater bone height compared to rh-BMP-2 in a collagen sponge carrier [28], whereas another systematic review stated that there were similar outcomes in terms of vertical bone gain, bone density, percentage of residual graft material, and percentage of vital bone, either with or without the use of rh-BMP-2 in sinus augmentation procedures [29]. In contrast, BMP-7, or osteogenic protein-1, still faces challenges related to non-specific activity and the need for precise delivery systems to optimize their regenerative potential. A split-mouth clinical study investigating sinus augmentation using BMP-7 reported significantly greater bone formation on the control side treated with bovine xenograft alone [30]. Thus, the use of BMPs in periodontal regeneration warrants many more studies to validate their role.

Platelet Concentrates

Platelets play a key role in hemostasis. They are rich in growth factors like PDGF, VEGF, IGF, and TGF-β. Various platelet concentrates, including PRP, P-PRP, L-PRP, and PRF, are actively employed in treatment protocols. Compared to synthetic biomaterials, autologous platelet concentrates are safer due to their biocompatibility and lower risk of immunogenic reactions. They also facilitate a more rapid vascularization and cellular infiltration, which are critical for periodontal regeneration [31]. However, studies show that their outcomes for recession coverage and GTR substitutes are not superior to traditional therapies. Due to variability in study design and technique, comparisons are challenging. While platelet concentrates benefit soft tissue healing, more work has to be done to clarify their mechanisms and clinical applications.

Enamel Matrix Derivatives (EMD) 

They are primarily composed of amelogenins, which are derived from Hertwig’s epithelial root sheath during tooth formation. An EMD aids in recruiting cementoblasts for the synthesis of new cementum and promotes the development of PDL and bone. A systematic review conducted by some authors compared clinical and radiographic outcomes after regenerative surgery and open flap debridement (OFD) for the treatment of deep (≥ 3 mm) intra-bony periodontal defects. The results reflected that both EMD and GTR were superior to OFD in terms of clinical attachment level gain; however, related to the other clinical and radiological outcomes, no significant difference was found between OFD, EMD, and GTR [32]. Yet another review on biologics-based regenerative approaches for periodontal soft tissue engineering found that consistent high-quality evidence supports the adjunctive use of EMD for root coverage procedures [33].

Stem Cell Therapy

Stem cell derivatives, which are self-renewing, include the periodontal, pulp, and mesenchymal stem cells (MSCs), which have shown significant potential in regenerating periodontal tissues. The MSCs can differentiate into osteoblasts, periodontal ligament cells, and cementoblasts [34]. This unique feature of the MSCs makes them ideal as regenerative adjuvants. They secrete a diverse range of bioactive molecules, including growth factors and cytokines. These secreted factors play a pivotal role in promoting tissue repair and regeneration. Growth factors like VEGF, fibroblast growth factor (FGF), and transforming growth factor-beta (TGF-β) help in the formation of new blood vessels within the surgical site, aiding in regeneration [35]. These cells also have very low immunogenicity as they do not express MHC class 2 molecules, and the MHC class 1 molecules are also expressed in low concentration. This characteristic enables MSCs to be safely taken from healthy donors and used in patients with periodontitis. Preclinical studies using stem cells have made use of animal models like rodents, pigs, and nonhuman primates like macaques. The analysis of periodontal regeneration using these models can be done using tissue samples for histological analysis, cone beam computed tomography (CBCT), and micro-computed tomography (micro-CT imaging) to assess the volume and architecture of the bone [36]. The immune response of the periodontal tissues after stem cell therapy could be assessed using both proinflammatory and anti-inflammatory cytokines as markers.

Limitations of stem cell therapy

The major limitation of employing animal models is the inherent heterogeneity of experimental models, and also the results obtained may not help in human studies due to the difference in the anatomy, immune response, and progression of diseases between animals and humans. As these studies are for a short period, the long-term safety and efficacy of stem cell therapy cannot be evaluated. Moreover, challenges are faced related to the standardization of protocols as well as the use of human stem cells in preclinical research due to regulatory and ethical concerns.

Three-dimensional scaffolds

Three-dimensional printing technology has revolutionized periodontal regeneration, enabling the creation of not only biocompatible membranes and scaffolds but also living cells and supporting elements within complex 3D functional tissues, a process known as "bioprinting." [37]. These scaffolds offer a potential solution for regenerating periodontal structures, but their success is often constrained by defect anatomy, angle, and remaining bony walls. Achieving a close adaptation between a block graft and the defect is challenging. To address this, 3D-printed biomaterials have been developed to fit precisely into defects based on CBCT assessments. However, for effective regeneration, 3D scaffolds must also have an internal microstructure that supports neo-angiogenesis, cell migration, and nutrient diffusion. Traditional scaffold fabrication methods lack control over pore structure, while techniques like electrospinning offer better structural control but present mechanical stability challenges.

Rasperini et al. published a landmark study using a human case that involved the treatment of an osseous periodontal defect with a 3D-printed bioresorbable patient-specific scaffold. Though the treated site was intact for a year, a month later the construct was exposed due to the low porosity and slow degradation rate [38].

Nanomaterials in periodontal regeneration

Therapeutic NPs, as a developing engineering solution for treating periodontitis, have become the focus of interest in recent times [39-42]. Nanoparticles are equipped with immunomodulatory, anti-inflammatory, antibacterial, antioxidant, and bone regeneration capacities. Silver (Ag) NPs have excellent antimicrobial activity against periodontal pathogens. The effect of silver NPs combined with ebselen, an anti-inflammatory agent, was assessed on *Porphyromonas gingivalis*, *Streptococcus gordonii*, and *Fusobacterium nucleatum* in planktonic conditions and as biofilms. The results showed that both the Ag NPs and ebselen not only had excellent antibacterial effects but also together declined the release of *Porphyromonas gingivalis*-stimulated inflammatory cytokines both in vitro and in vivo and reduced alveolar bone resorption effectively [43]. Wang et al. prepared a novel photothermal nanocomposite by encapsulating metal phenolic networks (MPNs) onto the surface of branched gold-silver (AuAg) NPs; AuAg@PC-Fe efficiently eradicated periodontal bacteria when exposed to 808 nm near-infrared light and also inhibited the nuclear factor kappa-B signaling pathway to treat periodontitis [44].

## Conclusions

The development of novel, bio-inspired materials designed to closely mimic the morphology of periodontal tissues at both micro and nanoscale levels is mandatory. Though the path is challenging, periodontal tissue regeneration strategies are evolving and are here to stay. The health of patients affected with periodontitis could be enhanced using these treatment modalities. The optimal concentration of growth factors for effective regenerative outcomes has to be validated. Tissue engineering methods using newer biomaterials like hydrogels, NPs, and biologicals have to be carefully evaluated by conducting longitudinal studies to enable their use regularly in patients. More importantly, the toxicity of NPs for a longer period on the crucial organs and tissues has to be assessed. Therefore, studies related to the absorption, distribution, metabolism, and excretion within the human body over extended periods have to be conducted. Finally, the use of these newer biomaterials should help in improving treatment outcomes for patients afflicted with the disease.
